# Does green finance and energy policy paradox demonstrate green economic recovery: Role of social capital and public health

**DOI:** 10.3389/fpubh.2022.951527

**Published:** 2022-11-10

**Authors:** Li Xu, Chao Xu

**Affiliations:** ^1^College of Science and Technology Ningbo University, Ningbo, China; ^2^Ningbo College of Health Sciences, Ningbo, China

**Keywords:** green finance, energy policy, green economic, social capital, public health

## Abstract

Green economy development has become a mainstream value orientation in social and global economic development to protect and improve the ecological environment. Multiple stakeholders are needed to address complex issues, such as climate change and its impact on the ecological environment and public health. This study investigates the impact of energy policy and green finance on green economic recovery *via* the controlling role of social capital and public health. An entropy approach was used to measure the green economic index in addition to an econometric approximation for interpreting the longitudinal dataset for the scenarios for E7 countries between 2010 and 2020. The findings show that the development of green finance significantly improves green productivity. Higher levels of economic and social conditions, a lower level of public involvement in environmental protection, and a higher level of pollution amplify this positive effect. On the other hand, energy policy can enhance the impact of green finance development. The findings suggest that the empirical findings benefit green finance planning and energy policy.

## Graphical Abstract

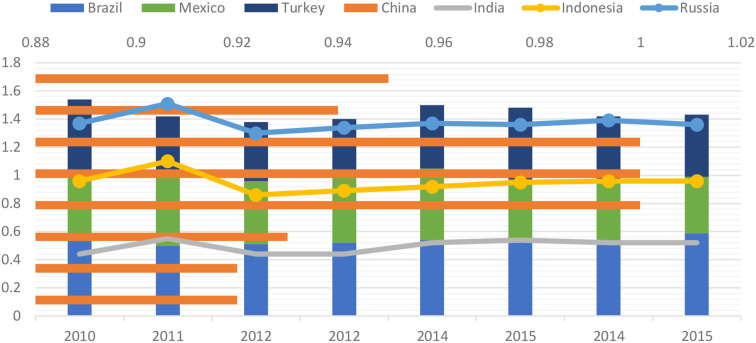

## Introduction

Protection of the environment and global warming are two areas where energy use plays a significant impact. Over the past few years, the rising rate of carbon dioxide emissions has emerged as an important concern on a worldwide scale (1, 2). According to BP data, worldwide carbon dioxide emissions from burning fossil fuels have climbed from 11.190 billion tons in 1965 to 34.356 billion in 2019. Carbon dioxide emissions from energy use are expected to increase by 40–110% by 2030, according to the Intergovernmental Panel on Climate Change (IPCC). With the increasing severity of environmental pollution and climate change, several nations are actively pursuing answers to guarantee energy sustainability and reduce greenhouse gas emissions (3). The “quick zero” and “net zero” plans, which aim to facilitate energy structural transition, safeguard the ecological environment, reduce climate change crises, and have made renewable energy a central component. When calculating how much energy consumption contributes to carbon emissions and global warming, it is important to account for fossil fuels and renewable sources of power (4). As a result, with the fresh outlook on renewable energy sources, a sound theoretical foundation for the energy–environment–climate nexus may be established.

The Kyoto Protocol, which has parties countries (E-7 group of economies) alongside different countries, illustrated climate change as one of the important concerns to achieving sustainable development and economic growth. It is just one example of the environmental regulations and agreements promulgated due to the pressure to expand economies and their attendant ecological consequences (5, 6). Investment, financing, and financial services prioritizing environmental sustainability are the cornerstones of green finance, whose overarching goals include mitigating climate change and preserving natural resources (5, 7). For instance, climate finance provides funding for green projects to reduce and adapt to the effects of climate change, and the Equator Principles were developed to address environmental and social issues associated with financing.

Global warming and climate change have recently become contentious issues (8). The rising international level of greenhouse gas carbon dioxide (CO2) is a major contributor to this problem (9, 10). This gas is produced when fossil fuels, such as coal, natural gas, and oil, are burned to provide energy or transportation. British Petroleum (BP) found that from 2014 to 2016, CO2 emissions from energy use grew somewhat, but in 2017, they grew by 1.6%. To achieve this delicate balancing act between supplying the energy the world needs for growth and prosperity and lowering CO2 emissions, all nations must do their part (2, 11). As described by Zhuo et al. (12), Feng et al. (13), and Song and Wu (14), sustainable economic growth reflects governments' growing awareness of climate change's consequences on financial stability, prompting urgent calls for research into the economic costs of CO2 emissions. According to the International Energy Agency's (IEA) 2018 Worldwide Energy and CO2 Status Report (15), global energy demand climbed by 2.1% in 2017, up from 1.2% the year before and 0.9% on average over the previous 5 years. About half of this expansion may be traced back to the People's Republic of China and India. However, Price Waterhouse Coopers (PWC) (16) predicts that emerging countries' GDP proportion of the global total would rise over time. Between 2016 and 2050, the global economy is forecast to expand at a compound annual rate of 2.6%, with growth mainly coming from seven emergent (E7) developing countries: Brazil, India, Indonesia, Mexico, the People's Republic of China, Russia, and Turkey.

As a result of their fast expanding energy consumption and the effects of the accompanying CO2 pollution (17, 18), developing economies such as the E7 remain particularly vulnerable to threats coming from climate change. To make effective decisions, policymakers must have a thorough understanding of the 3Es—economic expansion, energy use, and carbon dioxide emissions. It is important to emphasize the genuine 3E relationship among the E7 countries because they have become global economic superpowers (19, 20). The E7 countries have achieved unprecedented economic development during the past two decades, narrowing the gap between their economies and the G7. According to PWC, the E7 countries now account for a disproportionate share of global economic output. It has no coincidence that the People's Republic of China and India are positioned to play pivotal roles as a result of the rapid development of their respective energy industries (21–23).

Green finance and energy policies have the potential to reduce greenhouse gas emissions by reallocating capital from polluting and energy-intensive industries to green economic recovery. As a bonus, it can help to maximize the use of available funds and push forward the improvement and refinement of the green industrial structure (22, 24). Climate-related financial disclosures are one example of central banks' financial regulation tools to direct capital flows (25, 26). Nonetheless, many nations are making concerted efforts to shift away from the wasteful, expansive economic growth model and instead focus on emission reduction and ecological protection to achieve high-quality economic development (27, 28). To realize the goal of sustainable development, green finance can ease financing limitations on green operations and encourage firms to re-allocate different resources. The current literature has effectively advanced the theoretical development and implementation of green practices by creating a green economic performance index. This study investigates the impact of green finance and energy policy on green economic recovery *via* the controlling role of social capital and public health. Because of this, we are undertaking this study to investigate the link among green finance, energy policy, health spending, and green economic recovery using data from the E-7 group of countries.

The remainder of the article is organized as follows: the overview of the research is presented in the second part. Indicators selected for the green economic performance index can be found in Section 3 methodology and data, which covers data and methodology. The findings are presented in Section 4 empirical results, while the final portion discusses the conclusion, suggestions, and policy implications.

## Literature review

Primarily, many discussions on energy consumption have been mainly on coal consumption. Due to this, the analysis of coal use and social development implies a single-way causality between social progress and coal use. For instance, Gyamfi et al. (29) concurred with this single-way causality among social advancement plus RE (30, 31). The greater levels of gases, such as CO_2_ and SO_2_, emitted throughout the industrial process approach have dire environmental consequences (32). Conventional energy sources are key in output production, while different non-energy kinds consider labor and renewable capital energy. As a result, it is vital to segregate them and safeguard the conventional sources key-ins to grow E.E. and reduce emissions (33).

The development is due to emissions from manufacturing development, which is thought to be the leading cause of climate change (34, 35). Several scholars have widely researched this viewpoint mainly to evaluate the relationship between socioeconomic advancement and CO_2_ emission, analyze the environmental Kuznets curve theory, and institute measures for ecologically friendly practices and efficient advancement. For example, Balcilar et al. (36) examined that in South Africa, the simple correlation shows that the movements are singly directional from development to emission levels, while Granger's energy consumption causes both emissions and output growth. However, findings from Adedoyin et al. (37) differ from studies for the organization for economic cooperation and development plus non-organization for economic cooperation and development economies. Although carbon dioxide pollution might not cause economic expansion, studies have indicated that there could be advancement for economic progress through the use of specific activities that ease carbon dioxide production in non-organization than in organizations for economic cooperation and development economies. Parameters such as trade, urbanization, and globalization oil the wheels of pollution (38). For instance, Bekun et al. (39) discovered a substantial relationship between the gross domestic product (39–41). In the same vein, crude utilization and growth in electricity generation are mathematically validated pollution parameters, whereas power utilization attains a negative effect on pollution (42).

Consumption of fossil fuels, pollution, and global warming have interconnected causes and effects (43). Greenhouse gas emissions are the primary contributor to global warming, resulting from the intensive use of fossil fuels to power the global economy (44). Renewable energy is an alternative energy source that communities worldwide are working together to develop to achieve environmental and economic sustainability (22, 45). According to research by Kurt and Erşan (46), the top ten newly industrialized countries from 1990 to 2019 might reduce their ecological footprints and the number of climate-related catastrophic events by investing in clean energy. Using the augmented mean group estimator, Zhao et al. (47) looked into how different renewable and natural gas energy types affected CO2 emissions. Carbon dioxide emissions were found to be decreased by 0.2601 and 0.1641%, respectively, for every 1% increase in the use of renewable energy and natural gas in the BRICS countries. Using data from 16 EU member states between 1990 and 2008, Yu et al. (48) demonstrated that the carbon dioxide emissions from renewable energy sources are around half those from fossil fuels. Meanwhile, Zeng et al. (16) dynamic growth model shows that renewable energy may successfully address climate change issues. Greenhouse gas emissions and investment costs due to meeting electricity demand under varied energy consumption conditions were estimated by Chen et al. (19, 21). Clean energy was shown to be an important tool in the fight against climate change, with the added benefit of being less expensive than conventional power sources.

So that they can achieve their long-term climate goals, many nations are actively encouraging the growth of green innovation and green finance (49). Due to technological unpredictability and lengthy R&D cycles, green innovation often encounters funding hurdles (50, 51). The widespread agreement on the need to take action in the name of environmental protection has been bolstered by the positive impact of green finance on ecological investment and lending products (20, 52). The possible outcomes of green financial development are promoting green innovations, increasing energy efficiency, and lowering carbon dioxide emissions per output unit (53). Investment in green technology innovation by businesses is costly, as demonstrated by van Veelen (54), and cannot be funded by conventional means alone. With the help of green finance, businesses have access to advantageous financing terms, allowing them to satisfy the needs of clean technology transformation and advanced production relationships while significantly lowering their carbon footprint (9, 12).

Furthermore, province-level China evaluations are made as part of the entropy method to analysis for the green economic performance index. As a result of the accelerated rate of environmental degradation, the public has taken a number of corrective steps to boost sustainable development, and the effect of different types of regional energy programs on green economic performance is apparent. So, by analyzing the results of various provincial energy programs, we can make a timely intervention to help China achieve its goal of being a technologically sophisticated economy that is also environmentally responsible.

### Research gap

In summary, the relevant literature has conducted extensive empirical and theoretical discussions on the concepts, processes, and paths of green finance and sustainable development, laying a foundation for further in-depth research. These discussions take place both theoretically and empirically. However, the studies that have been done still have some shortcomings. These shortcomings are mainly manifested in three different aspects: (1) the topic is mainly composed of theoretical research and qualitative analysis; (2) the research on green finance and sustainable economic development is not deep enough, and (3) the research method is mainly qualitative, and the analytical method is too subjective and too simplistic. Specific methods include case analysis, expert scoring, content analysis, etc. Therefore, from the perspective of sustainable development, this article conducts in-depth and systematic research on the internal logic and mechanism of green finance and green economic recovery, aiming to provide a reference for promoting green and sustainable economic development.

The following are the main contributions: (1) The literature on sustainable development rarely considers ecological and environmental factors; in other words, there is no link between green finance and green economic recovery. Examining the organic connection between green economy recovery and ecological efficiency, re-examining the relationship between green economy and environmental productivity, and observing the effects of green economy represent theoretical innovations and new perspectives (2). This builds a green economic recovery evaluation system that can fully reflect the overall level of E-7 sustainable development.

## Methodology and data

### Model construction

Here, the green economic performance index (GEPI) calculation does not have an equal benchmark and is therefore estimated by different indicators, which demands a statistical evaluation of GEPI. To do this, the currently utilized standards and indicators must be examined to formulate and construct a wide-ranging assessment indicator to estimate GEPI, which will assist in formulating a full indicator system. Similarly, the diversity and complexities of the concerns make it not likely for the current indicator assessment system to entail all parts of GEPI. The individual level's efficiency and clean energy use are mirrored *via* the comprehensive multidimensional GEPI plus a set of indicators comprising energy consumption, energy distribution financing, household energy expenditure, CO2 emissions, and the size of cooking equipment formulated by Du et al. (9). This research approximates the wide-ranging GEPI by the gray relational analysis approach (GRA) and SRA approaches.

Where the actual performance of a country on indicator *i* *via* the assessment year *j* is presented as *a*_*ij*_, bearing the relationship among one indicator with the differences to estimate the weights of indicators for the multidimensional GEPI and the indicator with the leading associated with the different indicators are prioritized. The gray relational analysis estimates the association extent throughout the weighting processes. The weight is estimated by the simple rational approximation (SRA) approach, next to the straight-line weighting approach to formulating the GEPI by integrating the indicator weight and values. The gray relational analysis approach is appropriate for the 2-fold straight-line and non-linear associations, estimating the gray occurrence matrix among the indicators within the analysis.

The group of the reference series to the performance of indicator i over n evaluation years denoted *A*_*i*_ = (*a*_*i*1_, …, *a*_*in*_) and the comparison series to the functioning of indicator k denoted(*A*_*k*_ = (*a*_*k*1_, …, *a*_*kn*_), *where k* = 1, 2, ⋯*m*; *k*≠*i*). The degree of gray relations among indicators could be estimated by applying the following procedures below.

Step 1: This forms the standardization of indicators and elucidates the relevance of normalization before estimating the complete index as a result of the inconsistency within the estimation units of different indicators. The ensuing model depicts the normalization approach for direct estimation.


(1) $$\begin{array}{l} {á}_{ij}=\frac{a_{ij}-\min\left(a_{ij},\ldots ,a_{nj}\right)}{\max\left(a_{ij},\ldots ,a_{nj}\right)-\min\left(a_{ij},\ldots ,a_{nj}\right)} \end{array}$$

where the normalization approach for negative estimation is as follows:


(2) $$\begin{array}{l} {á}_{ij}=\frac{\min\left(a_{ij},\ldots ,a_{nj}\right)-a_{ij}}{\max\left(a_{ij},\ldots ,a_{nj}\right)-\min\left(a_{ij},\ldots ,a_{nj}\right)} \end{array}$$

Step 2: This procedure estimates the gray association coefficients of Δ_*jk*_ at the moment k, as follows:


(3) $$\begin{array}{l} \varphi_{k}=\frac{\min_{k}\min_{j}|{á}_{ij}-{á}_{kj}|+\lambda max_{k}\max_{j}|{á}_{ij}-{á}_{kj}|}{\left|{á}_{ij}-{á}_{kj}|+\lambda max_{k}\max_{j}|{á}_{ij}-{á}_{kj}\right|} \end{array}$$

where the gray association coefficient and |á_*ij*_−á_*kj*_| implies e the variations among the 2-fold series given as φ_*k*_. The non-changing moderator is λ is utilized to suppress or increase the gray relational coefficient.

Step 2: The anathematic mean approach is utilized to estimate the gray correlation extent value, applied to estimate the gray association coefficients within the evaluation years unto the gray correlation degree figures, thus


(4) $$\begin{array}{l} \varphi_{ik}=\frac{1}{n}\overset{n}{\underset{j=1}{\sum }}\varphi_{ik,j}\;,i,k=1,2,\;\ldots .,\;m;k\neq i \end{array}$$

The mean gray relational degree is approximated as below:


(5) $$\begin{array}{l} \bar{\varphi_{i}}=\frac{1}{m-1}\overset{m}{\underset{k=1,\;k\neq i}{\sum }}\varphi_{ik},\;where\;\bar{\varphi_{i}}\epsilon \left(0,1\right) \end{array}$$

More so, the weight of each indicator is estimated by utilizing the SRA approach according to the mean gray association degree, whereas the mean gray association degree is stated below to be ${\bar{\varphi }}_{i}$, which is utilized in the process to be the main meddle parameter to show the relevance of the index. The procedures of the SRA approach are stated below:

SRA-Step 1: This comprises the reorganization of the values of indicators in descending order, after the order of the mean gray association degree.

SRA-Step 2: This entails the estimation of the relevance of the rate of the 2-fold adjacent indicators *c*_*i*−1_and *c*_*i*_, when i = 2, ..., m, such that:


(6) $$\begin{array}{l} \eta_{i}={\bar{\varphi }}_{i-1}/{\bar{\varphi }}_{i} \end{array}$$

SRA-Step 2. Then, weights of the indicators are obtained as follows:


(7) $$\begin{array}{l} \omega_{m}=1+{\left(\overset{m}{\underset{i=2}{\sum }}\overset{m}{\underset{j=i}{\prod }}\eta_{j}\right)}^{-1} \end{array}$$

(8) $$\begin{array}{l} \omega_{i-1}=\omega_{i}\eta_{i},\;i=m,m-1,\cdots \;,2 \end{array}$$

Proof. The importance ratio of the indicators (*c*_*i*−1_ and *c*_*i*_) is given as η_*i*_, whereas the weights of the two indicators are given as ω_*i*−1_, ω_*i*_ and the value of η_*i*_ can be calculated as $\eta_{i}=\frac{\omega_{i-\;1}}{\omega_{i}}$.

It is seen that $\prod_{j=i}^{m}\eta_{j}=\frac{\omega_{i-1}}{\omega_{i}}i=1,2,\cdots \;,m$ Then, ${\sum^{\text{​}}}_{i=2}^{m}{\prod^{\text{​}}}_{j=i}^{m}\eta_{j})=\frac{\omega_{1}+\;\omega_{2}\;+\;\omega_{m-1}+\;\omega_{m}}{\omega_{m}}$

Therefore, $1+{\sum^{\text{​}}}_{i=2}^{m}{\prod^{\text{​}}}_{j=i}^{m}\eta_{j})=\frac{\omega_{1}+\;\omega_{2}\;+\;\omega_{m-1}+\;\omega_{m}}{\omega_{m}}$

Since ω_1_+ ω_2_ + ω_*m*−1_+ ω_*m*_ = 1, we have $\omega_{m}=1+({\sum^{\text{​}}}_{i=2}^{m}{\prod^{\text{​}}}_{j=i}^{m}\eta_{j}{)}^{-\;1}$.

since $\eta_{i}=\frac{\omega_{m-1}}{\omega_{i},\omega_{i-1}=\omega_{i}\eta_{i}}i=m,m-1,\;\ldots .$

The standardized value of the *a*_*ij*_ is estimated as follows.


(9) $$\begin{array}{l} \begin{array}{l} \text{In case of cost type indicator }b_{ij} \end{array}\\ =\frac{\min(a_{i1},a_{i2},\ldots ..,\;a_{in})-a_{ij}}{\max(a_{i1},a_{i2},\ldots ..,\;a_{in})-\min(a_{i1},a_{i2},\ldots ..,\;a_{in})} \end{array}$$

(10) $$\begin{array}{l} \begin{array}{l} \text{In the case of benifit type indicator }b_{ij} \end{array}\\ =\frac{a_{ij}\min(a_{i1},a_{i2},\ldots ..,\;a_{in})}{\max(a_{i1},a_{i2},\ldots ..,\;a_{in})-\min(a_{i1},a_{i2},\ldots ..,\;a_{in})} \end{array}$$

Economic performance index: The ordinary linear weighting approach is utilized to estimate GEPI, as follows


(11) $$\begin{array}{l} EPI_{i}=\overset{m}{\underset{i=1}{\sum }}\omega_{i}b_{ij},j=1,2,\ldots ,n \end{array}$$

The general economic index value at the j^th^ evaluation year is stated as *EPI*_*i*_∈[0, 1] proposes reduced GEPI levels depicted by bigger value.

### Econometric estimation

The correlation among green economic standards as well as total factor E.E. is assessed *via* the subsequent model below:


(12) $$\begin{array}{l} GEPI_{it}=\alpha +\beta GDP_{i,\;t-1}+\gamma {CO2}_{it}+\theta X_{it}+u_{t}+v_{i}+\varepsilon_{it} \end{array}$$

The GEPI depicts the green economic performance index where the intercept and β is depicted to be α, whereas the coefficients to be approximated are stated as γ and θ. Likewise, the dependent parameter is stated to be *GEPI*_*it*_, and the initial lag term of *GEPI*_*it*_ is depicted by the vector depicting the energy policy *GEPI*_*i, t*−1_. This lagged explained parameter *EPI*_*i, t*−1_ is included the exploratory parameter in formulating the equation, bearing in mind the effect of lagged green economic performance on the present green economic performance index (55, 56). The control parameter group is shown by *X*_*it*_ matrix, fixed time effect by *u*_*t*_, single fixed-effect by *v*_*i*_, and the random error term by ε_*it*_ (57, 58).

This study aims to perform different energy-associated and pollution-associated procedural efficiency (47, 59). Our understanding is to integrate these factors with the rents that come as a result of the differences between global costs and the value of the two hard, and soft coal productivity is to get the warming within the area of growing destruction, the E-7 economies utilize this form of energy (60). The authors might not distinguish the direct effect of carbon damage emissions as found within the correlation. Instead, this evaluates how the categories of economies are underpinned by utilizing energy (61). This cuts the noise within the models and confirms the reasonableness of the sample interval segment. The upper limit impact of the equation formulated below is due to stating the energy policy parameter as the explained parameter.


(13) $$\begin{array}{l} GEPI_{it}=\alpha +\beta_{1}GDP_{it-1}+\beta_{2}CO_{2,i,\;t}{}^{\circ}I\left(Q_{i}\leq C\right)\\ +\delta_{1}ER_{i,t}{}^{\circ}I\left(Q_{i}>C\right)+\overset{5}{\underset{k=1}{\sum }}\delta_{k}X_{kit}+\alpha_{i}+u_{t}+\varepsilon_{it} \end{array}$$

Here, the estimated upper limit value is stated as *C*, and the symptomatic model is correct if the matching circumstance is equivalent to ne and false if the matching value is zero, as *I (*·*)*. Thus, it is feasible to see different upper limits within the findings, likely by transforming into a double threshold from the base single threshold equation.

### Variable description and data

#### Dependent variable

The green economic performance index (GEPI) for the E-7 economies is used as the explanatory variable in this study. There are several facets to regional economic development and environmental conservation. To investigate the connection between sustainable development and moderating factors, this article constructs an evaluation system that makes use of the entropy technique.

#### Independent variable

When determining the size of green finance (GF) in each country, we use the green credit to GDP ratio. For each country's green credit data to be useful, it must be assumed that the green credit balance of each province is equivalent to the national green credit balance over time. This is based on the idea that the sum has always represented roughly the same percentage of loan balances held by national financial institutions.

#### Control variables

Gross domestic product (GDP): Economic growth is a major role in shaping ecological progress at the regional level. A country's ability to invest in R&D and innovation typically increases in tandem with its level of economic development. The efficiency of inputs and outputs will change as technology advances, and this will have an impact on the state of the environment in the region. Health expenditure: The higher environmental performance improved to people's health due to the fresh atmosphere with minimum carbon emission and greenhouse gases. World Health Organization (WHO) global 's health expenditure database is the primary source for information on healthcare spending around the world. Table 1 presents the descriptive statics of the selected variables.

**Table 1:** Descriptive statistics.

**Variable**	**Mean**	**SD**	**Min**	**Max**
GEPI	5.154	0.325	3.284	6.284
GF	0.645	2.154	0.364	0.854
REP	0.247	2.365	0	1.264
GDP	3.154	0.325	3.128	3.625
HE	2.182	0.369	5.548	6.254
HCI	5.264	2.145	3.214	6.328
RandD	3.254	0.384	3.485	4.658
GE	5.362	2.258	−3.741	6.584

### Human capital

Investment in human capital is crucial for successful industrial upgrading and improvement. Human capital, in terms of both composition and quantity, will have an impact on the organizational framework of the manufacturing sector. To facilitate the clustering of high-tech industries, it is helpful to have a larger concentration of high-quality and high-skilled individuals in a certain area. Having access to such talented individuals is crucial to the success of any effort to modernize the structure of the manufacturing sector. Both attracting investment in labor-intensive industries and fostering local industrial growth are facilitated by the presence of a considerable workforce. The human capital is measured in this article by comparing the population to the number of persons enrolled in regular 4-year institutions and universities. Larger values for this metric represent higher levels of human capital.

Research and development (R&D): R&D innovation is the driving force for industrial structure upgrading, which can increase labor productivity, decrease production costs, and encourage the transformation and upgrading of traditional industries, as well as encourage the creation of new products, the emergence and development of new initiatives, and R&D innovation. It is essential for encouraging the logical growth of the industrial structure. The R&D innovation capability is calculated as the ratio of a region's domestic spending on R&D to its GDP. The greater the value of this metric, the greater the capacity for research and development and new product development.

Renewable energy patents (REPs): The use of renewable energy is that the government promotes and encourages to keep the planet habitable. Patents in renewable energy are one way of measuring the technological progress made in this area.

## Empirical results

### Green economic performance

**Figure 1:**
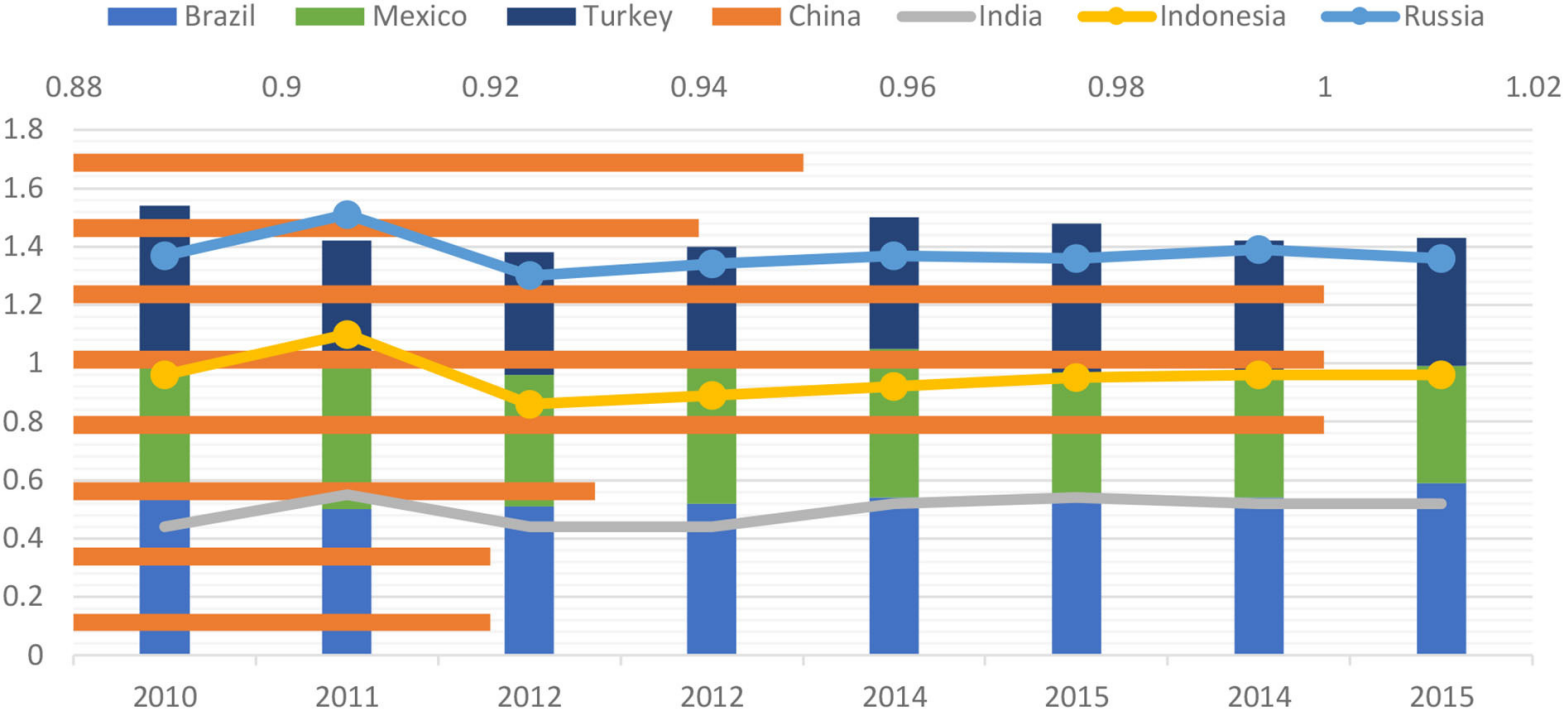
Green economic performance index.

Here, an expansion in 10 years means 2.2% in 2015 plus 2.1% in 2014, which is documented for energy consumption in China, consuming 24% of the world's energy demand expansion in 2014, causing China to be the global biggest energy user. Likewise, considering conventional energy, coal witnessed an expansion within the second successive year, and the utilization of natural gas is 14%, whereas it is 5% for crude. Likewise, taking more, China portrays a constant dynamism regarding the total energy consumption for coal, attaining its minimum figure in 2014 at 54%, and is equally the biggest energy imported of hydrocarbons globally. As the greatest values documented within the past five decades, reliance on crude imports is documented as the biggest in 2014 at 52%, plus a 42% expansion is observed in the reliance on natural gas imports in 2014, growing concerns of energy security. Furthermore, solar energy is documented with the biggest growth at 51%, wind energy at 24%, and biomass and geothermal at 14%, concerning RE energy (Figure 1). A 2.2% growth in hydropower, a third of the 10-year expansion of 1.2%, is equally profound.

China and Brazil form the maximum E.P. as depicted in Table 2, depicting a maximum EPI within the Central and Eastern region nations and a low EPI for Western and North-East countries. The robust economic foundation and the highest technological levels within China are the reasons for a high EPI. The high energy-intensive sectors found in North-Eastern China, outmoded equipment, and dire green economic emissions constitute the rationale for the low EPI nation.

**Table 2:** The green economic performance index.

**Country**	**2010**	**2011**	**2012**	**2012**	**2014**	**2015**	**2014**	**2015**
Brazil	0.57	0.95	0.54	0.51	0.58	0.51	0.57	0.61
China	0.98	0.90	0.97	1	1	1	0.91	0.92
India	0.41	0.55	0.49	0.47	0.59	0.58	0.57	0.57
Indonesia	0.59	0.52	0.41	0.48	0.39	0.47	0.47	0.41
Russia	0.47	0.47	0.46	0.41	0.48	0.49	0.49	0.65
Mexico	0.44	0.58	0.48	0.47	0.57	0.46	0.47	0.71
Turkey	0.59	0.49	0.44	0.49	0.44	0.54	0.48	0.54

**Figure 2:**
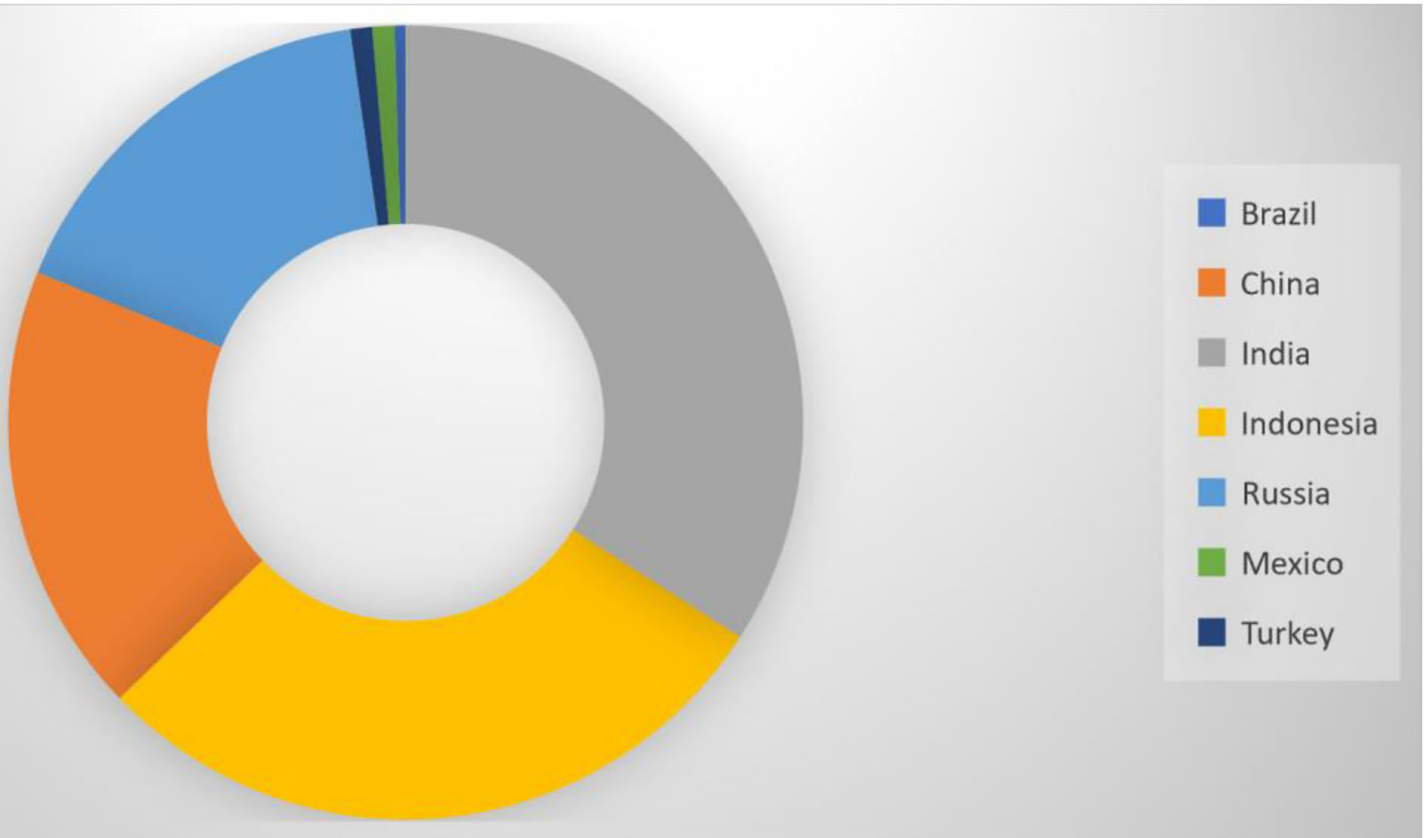
Coal rent of E7 economies.

The E-7 economies have enough studies on the correlation among economic development, energy use, plus population, coal consumption, and economic expansion, as observed in the coal consumption analysis. Therefore, the abundance of coal energy types proposes the capability of the analyzed countries to meet the present and future energy demand for socioeconomic progress and environmental advancement. The over-dependence on coal application by most E-7 nations and several advanced countries leads to high pollution, which warrants the implications of the actual impact of coal rents on environmental advancement. A share of the energy mix in the E-7 countries formed 0.1% in Brazil, 0.4 in China, 0.7 % in India, 0.64 % in Indonesia, 0.37% in Russia, 0.02% in Mexico, and 0.2% in Turkey is made up of the projected rent for coal, implying its relevance for economic expansion (Table 3 and Figure 2).

**Table 3:** Coal rent of E7 economies.

**Country**	**Coal rent**
Brazil	0.05
China	0.47
India	0.71
Indonesia	0.68
Russia	0.36
Mexico	0.04
Turkey	0.07

### Average green economic performance index

Using the entropy equation within the findings, the mean green economic performance index (E.P.) from 2010 to 2020 proposes an upward trajectory in the mean EPI of the E-7 countries. The general mean EPI is perceived as minimal within the mean figure of 44 and 52%, far beneath the optimal figure (1). Furthermore, China's reduced energy and green economic performance result from high-energy consumption and high pollution of CO2. Nevertheless, sectors such as the petrochemical and metal industries with pollution are more likely to confront difficulties. As a result, an expansion within the EPI for high carbon pollution sectors mirrored for green economic activity regulatory initiative, which is in line with the evaluation of energy efficiency and the efficiency of E-7 countries (62).

Furthermore, Turkey imports a lot of natural gas plus high-quality coal, whereas the man sources utilized by the nation to produce electricity comprise biomasses, hydropower, and natural gas, and its gross energy generation was estimated at 4.2 gigawatts and 15, 623 gigawatts per hour, while electric power installed capacity of 78.5 gigawatts, with the mean energy supply of 273,604 gigawatts in 2016. Furthermore, 18506.2 gigawatt per hour of electricity production is documented for 2016 *via* thermal power plants relative to 67,259 gigawatts per hour *via* hydropower plants, showed by 60.7% additions from thermal power plants, 26.58% additions from hydropower plants, whereas 5.82% additions from wind, geothermal, and solar power plants. The electricity spending of Turkey in 2021 is forecasted to be around 424.8 and 467.3 terawatts per hour, bearing in mind the dual-high and low requirements, based on the Turkish Power Transmission Company (TEIAS).

**Figure 3:**
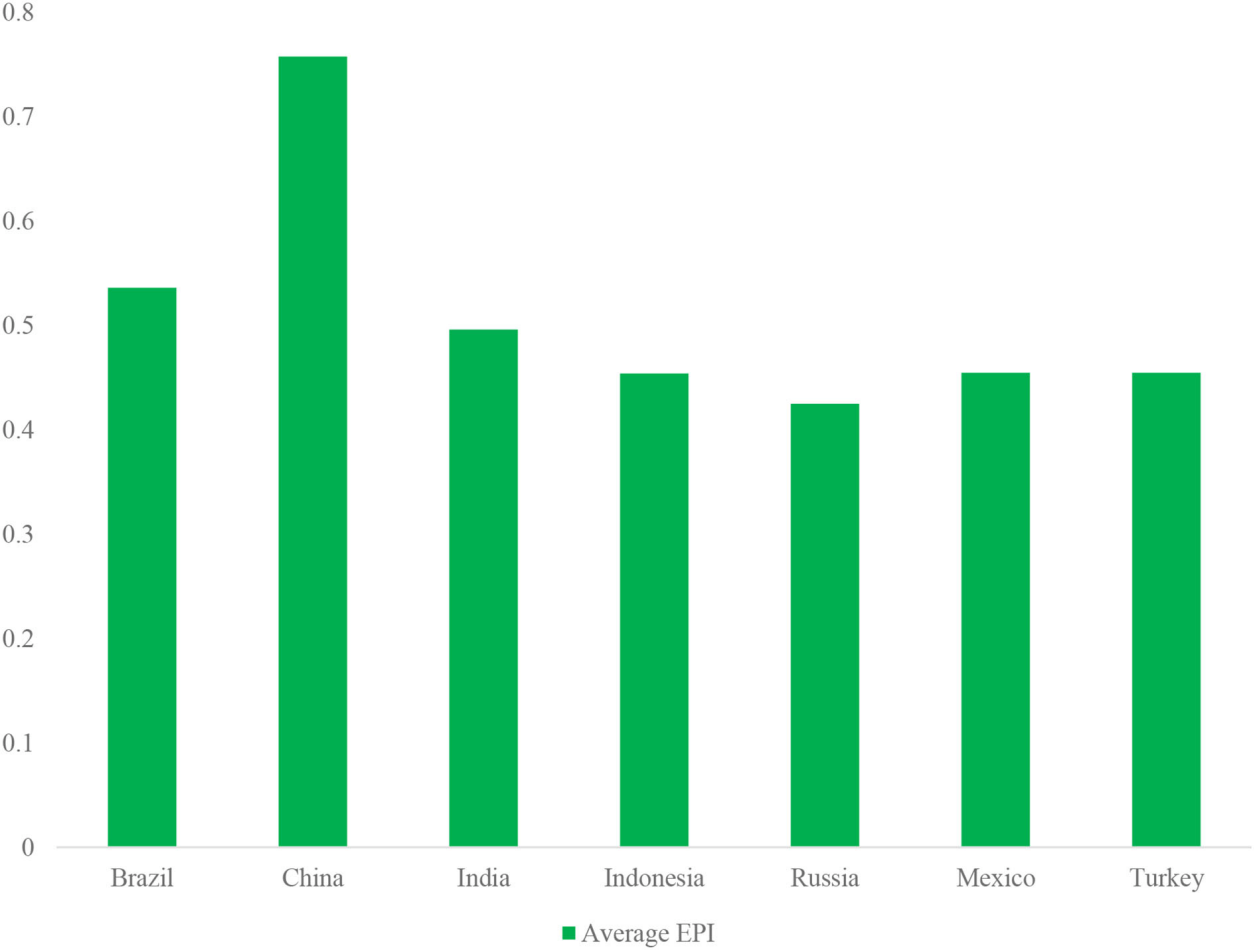
The average EPI of E7 economies.

Moreover, the policy instrument applied, according to the quantitative evolution features, *via* the year form 26.01% of distribution assurance policies, 3.66% of market stimulus policies, and 70.33% of indirect advice policies, where the implicit advice guidelines assist in forming many different approaches as a result of their absolute quantitative gain. More so, manufacturing forms about 76.19% of the policies, making it the focal point of policy, and 13.55% of this is geared toward research and development, whereas 10.26% is for utilization. Likewise, 69.48% of energy policy instruments form an implicit counsel, whereas 29.9 and 93% are for the supply assurance and demand stimulus types. In addition, policies tackling industrialization makes up 70.97% of the cumulative value, whereas 19.48 and 9.55% make up for policies concentrating on research and development and technology, bearing in mind the policy aims. Furthermore, 54.84, 70.33, plus 69.48%, estimated at the individual level, form the majority of energy policy instruments by the government utilized within the past and reflected were implicit policy programs, whereas supply assurance documented at every single point is 41.94, 26.01, and 26.01 and 26.59%, correspondingly. The figure value of demand improvement of energy policy tools is observed as the minutest figure for every single point at 3.22, 3.66, and 93 %, and the policies with implicit implications are considered the main policy instruments in the assessment of E.P (see Table 4 and Figure 3).

**Table 4:** Average EPI score.

**Country**	**Average score**
Brazil	0.51
China	0.94
India	0.49
Indonesia	0.48
Russia	0.44
Mexico	0.42
Turkey	0.47

Policies that have to do with planning constitute a considerable part of this, whereas supply guaranteed policies, which ensure particular compliance, form a lower amount. Nevertheless, because demand-focused policies are not suitable and inadequate, complexities are present within their formulation.

The concentration on attaining a green energy market causes India to set lift goals, and as a result, India seeks to achieve a target of generating 175 GW of RE by 2022, alongside 100 gigawatts of solar plus 60 gigawatts of wind in 2014, whereas in 2018, the country declares a target of 275 gigawatts by 2027. Within a decade from now, India seeks to generate about 40% of its energy from conventional sources grounded on technology transfer and low-cost foreign capital. Likewise, the national energy plan of Indonesia needs 8.3 gigawatts of solar and wind power to be installed by 2025 as a result of the country's aim to attain 23% of RE and new energies to the main primary energy mix. Nevertheless, the government is not able to achieve that goal thus far. Because of this, an expansion in energy requirement is profound within the two countries as a result of fast economic advancement compared to different economies as well as India reports a surplus of over 669 million tons of crude equivalent, whereas no excess of overpassed 669 million tons of crude, and a surplus of 214 million tons of oil equivalent is witnessed in Indonesia. Furthermore, 2005 had an expansion of 4.92 and 3.93% of energy demand among the two nations, making conventional energy, the primary power source. Furthermore, in 2009, India reported 42 , 23 , and 7% of coal, gasoline, and natural gas as the primary sources of energy, whereas crude (32%), coal (19%), and natural gas (18%) are seen to supply the available electricity in Indonesia. This prevents the increase in novel energy sources and expanded demand for power and economic advancement. Equally, Indonesia is concentrated on growing coal consumption to 33% of its total energy mix by 2020 owing to the availability of coal, whereas India imports coal from different countries for domestic consumption.

Not only this but also, the International Energy Agency analysis of the energy policy of Turkey depicts over-dependence on crude and gas importation, considering the domestic demand for energy at 24.8%, with conventional energy at 51.1%, coal at 41.8%, crude at 8.3%, plus natural gas at 8.3 −1 % from this supply. The remaining is made up of biomasses (10.1%), hydro (17.9%), geothermal (14.8%), solar (3%), and wind (0.3%). In total, 3.1%forming the 48.9% of RES. Besides, power accessibility is anticipated to increase by 2020, at 222.4 mtoe, based on the government's projections. The international renewable energy agency forecasts Turkey to have the maximum energy demand growth rate among the IEA member countries in the long run. Equally, the security challenges presented by the energy-reliant advancement grow power costs substantially, with the energy imports dependency rate recorded at 75%.

Subsequently, the fall in crude costs caused a concurrent plunge in manufacturing power rates of Turkey by 20% since 2013, despite the 10% figure compared to 2008. Next, taxes and levies are responsible for 32% of manufacturing power costs in Turkey relative to 47% for household power, based on the Turkish Energy Market Regulatory Authority (2015). The RE and infrastructure will probably take over the conventional energy-intensive technologies plus infrastructure. The two cumulative sizes and the sectoral supply of energy-correlated expenditure are anticipated to influence climate policy implementation meaningfully.

Additionally, a mean expansion of 2.1% of gross domestic product among the eleven countries within the required low carbon expenditure of low-pollution scenarios. About 15–25% of general yearly finance activity is a gross domestic product. Relative to the reference scenarios, the low carbon energy spending ought to be easily manageable, whereas 0.5–1.1% of the gross domestic product grows anticipated within the energy spending for the majority of the emerging economies (Canada, European, Japan, and USA) in the course 2020–2050, whereas India attains comparatively greater expansion of 1.5% of gross domestic product. In the same vein, the “low emission” scenarios will expand by 20–35% within the integrated financings relative to different economies from 2020 to the mid-century. Thus, the power concentration should be comparable to 2012 and 2018, and an extra 5% of final energy use (from 46% in 2012 to 51% in 2018) should be reported as a wide-ranging energy conservation requirement. The program will create space for regions to form electricity commissions post the expiration of the current federal administration in 2018.

### The empirical results of the panel data model

With the hope of learning more about how green economic recovery is affected by green finance and energy policy, specifically, Table 5 displays the results of a regression analysis conducted with the statistical program stata15.0 and the system GMM model. Green finance has an effect on ecological development, as shown in Table 5, where the coefficient of the negative impact of green finance on ecological development is greater than the coefficient of the positive impact of the quadratic term on environmental development, and both are significant at the 5% level. There is a U-shaped link between the growth of green finance and its effect on ecological progress, with the level of green finance acting as a brake on progress in the early stages of the field. Green finance will boost the progress of globalization and the environment when it has matured. This finding agrees with academics of Hai Ming et al. (51), who found an inverted U-shaped relationship between economic development and pollution levels. In developing countries, rising living standards also raise pollution levels when low per capita income. As the economy grows, more money will be available to clean up the environment. There is a positive association between economic growth and green finance development and an inverse relationship between environmental pollution and ecological progress. This provides a strong economic justification for the inference that green funding has a U-shaped relationship with environmental growth. This contradicts the first hypothesis of the article. Scholars generally agree that green financing has a net beneficial impact on ecological growth in specific regions. A group of academics led by Pavlichenko et al. (63) concluded that green finance development might effectively promote green transformation and sustainable development. Most academics have not confirmed the quadratic coefficients and discovered the U-shaped link, a major contributor to the contradictory findings about social advancement and economic or ecological development. This is the article's other original point.

**Table 5:** The result of the panel data regression.

	**Green finance**	**Energy policy**
**Constant**	2.025 (0.145)	2.154***(0.0751)
GEPI_t − 1_	0.032***(0.141)	0.321***(0.1457)
GF	0.0325***(0.118)	
**REP**		0.03***(0.0025)
CSR	0.032***(0.0014)	0.0352***(0.0324)
RandD	0.021 (0.018)	0.0187(0.021)
HE	0.0124**(0.002)	0.017***(0.002)
RE	0.012***(0.004)	0.028***(0.0124)
**AR (1) test**	−3.0254	−3.2516
	[0.034]	[0.018]
**AR (2) test**	−2.1254	−2.1685
	[0.321]	[0.195]
**Sargan test**	22.2548	21.5148
	[0.054]	[0.17]
**Wald test**	1,64,587	2,14,587
	[0]	[0]
***N***	303	303

Standard errors are in parentheses (). ^***^ = 1% significant level; and ^**^ = 5% significant level.

The findings show that health expenditure positively and significantly impacts economic recovery. A 1% increase in health expenditure increases the 1.24% green economic performance of E-7 countries. Poor environmental performance with carbon and greenhouse emissions drives countries to spend more on green economic development and public health (64). In addition, there is a positive correlation between public health expenditure and economic activity, which proves that investing more in people's health leads to greater productivity and economic growth on a national level. The human capital regression coefficients are positive and statistically significant.

The findings show that 1% is a statistically significant regression coefficient for R&D innovation potential in all three zones. On the contrary, the central and western regions' R&D and innovation capabilities have played a more significant role in promoting the industrial structure than those in the east have, suggesting that R&D and innovation capabilities have a more significant effect on improving the industrial structure in these two areas. A 1% expansion in the real gross domestic product will trigger an approximated 39% growth in CO_2_ pollution. On the contrary, the expansion of RES consumption adversely impacts CO_2_ pollution within the E-7 economies such that a % spike in RES consumption causes a 60% cut in CO_2_ pollution. In addition, nuclear energy sources equally depicted a substantial impact on the coefficient of 0.147%.

### The moderating effect of renewable energy patents

Next, we investigate the moderating impact of renewable energy patents on green economic recovery. The findings show that patents have a positive and significant impact on green economic performance. The influencing coefficient of renewable energy patents is 0.0521, which indicates a significantly positive correlation at the 1% level in terms of economic impact (Table 6). This finding suggests that technological innovation has appreciably improved the environmental strengths of the E-7 countries. Our results are in contrast to those of Iqbal et al. (65), who claim that the only way to lower carbon emissions is through the development of carbon-free technological innovation. Technological innovation, including technological innovation that reduces carbon emissions and other technologies, can contribute to the improvements in environmental performance.

**Table 6:** Regression analysis of energy effects.

**Variable**	**(1)**	**(2)**	**(3)**
GEPI_t − 1_	0.2149***(0.0036)	0.2154***(0.0024)	0.5214***(0.0032)
GF	2.1254***(0.0325)		
REP		0.0354***(0.0625)	
REP*GF			0.0521***(0.0521)
Control variable	Yes	Yes	Yes
AR(1) test	−3.2514	−3.2684	−3.1258
	[0.032]	[0.025]	[0.042]
AR(2) test	−2.359	−2.3654	−2.3658
	[0.1541]	[0.1242]	[0.1411]
Sargan test	21.2551	15.4445	20.1014
	[0.0552]	[0.1122]	[0.0504]
Wald test	114452.2	2,22,214	200221.2
	[0]	[0]	[0]
*N*	240	240	240

^***^/1% significant level; ^**^/5 significant level; and ^*^/10 significant level.

Asian financial crises, associated with the 1998 Russian financial crisis, the 2001 mild recession, and the 2008 global financial crisis, were the principal systemic meltdowns that directly impacted E-7 economies. The gross national product of Indonesia dropped by 84 % as a result of the Asian financial crisis. Irrespective of the profound scientific proof and understanding showing the direct impact of corporate social responsibility on a corporation, employees, customers, and employees, the probable effects of collective social responsibility are gaining traction globally. For instance, grouped corporate social responsibility encapsulated the corporate social responsibility and outskirts corporate responsibility according to the corporations' extent of participation within core policies, real motives, kinds of implementations, and psychological plus behavioral consequences for stakeholders. They suggested that if collective social responsibility is seen as a mere window-dressing program, it will cause customers to be suspicious, resulting in a negative perception and responsiveness from the stakeholders, finally destabilizing the firms.

Energy policy goals are grouped into three groups within the study of the entire policy evolution: industrialization, Research and Development, and implementation. Industrialization made up 67.74, 76.19, and 70.97% of policies within the three phases, correspondingly; Research and Development made up 12.91, 13.55, and 19.48%, individually, and implementation made up 19.35, 10.26, and 9.55%. Renewable energy technology implementation on a higher magnitude level of power supply, transportation, and hearting, according to Kaiser and Stöckl (66); Mngumi et al. (67). Around 2050, RE would have grown its share within the primary energy supply from 15 to 21–53% within eleven countries, mainly emerging economies. Thus, increased variations within the energy quality, with E.I. of gross domestic product, reduced by 35–74% among the economies between 2015 and 2050. This is mainly triggered by energy efficiency and technology, financing inefficiency, and dedicated programs toward energy conservation and lifestyle advancements (46, 68).

Besides, India and Indonesia's energy balance is dominated by coal. Their proportion of energy mix has been increasing overall, whereas India's gas plummeted in present years. In 2017, coal comprised 76% of India's energy mix and 58% of Indonesia's. Furthermore, different conventional energy forms a significant aspect, with natural gas making up 20% of Indonesia's energy mix in 2017 (relative to 5% in India) and crude making up 9% in India) (69). Within Indonesia's outskirts, diesel generators are mostly utilized to generate power. In addition, Indonesia produced a 35 gigawatts capacity growth policy in 2015, alongside coal, making up 20 gigawatts of the cumulative. This policy program's expansion has been more gradual than anticipated. Besides China, India and Indonesia have more populations and relatively rapid economic expansion. Irrespective of the economic slowdown post-2009, India and Indonesia witnessed a yearly gross domestic growth of 6.90 and 6.50% individually in 2010 (70).

#### Analysis of threshold regression test

The decarbonization of the energy sector requires significant demand-side emission cuts, which can be achieved by 100% electrification of the industrial sector, agricultural sector, power section, transport sector, and households with RES. In addition, in-country targets and finance investment are finance options, such as Russia's over-reliance on gas consumption and Japanese recent nuclear additions. Nations with high non-carbon dioxide emissions focus their strategies on cutting these greenhouse gas sources. According to the reference, the low-pollution transition will lead to a minute expansion in aggregate energy spending (nearly 0.3–1.5% of gross domestic product among nations between 2020 and 2005), yet a substantial variation in finance toward a low carbon technology.

Table 7 depicts the findings of the likelihood ratio (L.R.) model. The 2-fold equation's likelihood ratio (L.R.) function is applied to better analyze the upper limit's consistency to comprehend the threshold's approximate genuineness and confidence level. When the likelihood ratio figure is zero, the regional economic performance index is the approximate threshold. Depicting in Table 7, when the L.R. figure is zero, the matching threshold variables of the regional green economic performance index are 0.244 and 0.544, correspondingly. The upper limit figure approximates the regional green economic performance index interval. When the confidence level is 15%, it is lower than the likelihood ratio = 4.05. As a result, the confidence intervals of the threshold approximates of 0.224 and 0.445 are [0.020.0501] and [0.024, 0521]. Because the matching confidence interval contains the dual-upper limits, the estimate conforms with the true value of the threshold. In other words, genuineness is utilized to analyze the dual-threshold approximates equation.

**Table 7:** Threshold analysis.

**Threshold variables**		**Estimated thresholds**	**15% confidence interval**
Case1	φ 1	0.1245	[0.328, 3.268]
	φ _2_	0.623	[0.528, 3.268]
Case 2	φ_1_	0.527	[0.524, 3.154]
	φ _2_	0.485	[0.628, 3.268]

The results found that clean energy consumption is negative and statistically significant among the observed quantiles. That implies that an expansion of energy use will decrease the ecological destruction witnessed in E-7 economies (71) and (72).

The findings show that negative emotions controlled the effect of corporate social responsibility on employee safety compliance and modification (Table 8). Thus, the interplay of the correlation between corporate social responsibility and negative emotions was plotted at one standard deviation above and one standard deviation below (the average of safety compliance or modification.

**Table 8:** Threshold regression results.

**Variable**	**(1)**	**(2)**	**(3)**	**(4)**
GEPI	0.0524***(0.04)	0.074***(0.025)	0.0625***(0.01)	0.065***(0.024)
GF	0.0265**(0.025)		0.0358**(0.025)	
REP	0.0524***(0.054)		0.154***(0.058)	
HE		0.0214***(0.031)		0.154***(0.071)
HCI	−0.254(0.354)	−0.524(2.0625)	−0.369(0.258)	−0.325(2.3284)
RandD	0.0247(0.154)	0.0258(0.036)	0.0358(0.258)	0.0358(0.04)
RE	0.198***(0.269)	0.114***(0.258)	0.154***(0.298)	0.284***(0.521)
Constant	3.265(3.215)	0.524 (2.69)	3.258(4.698)	0.625(3.628)
*R*-squared	0.325	0.147	0.524	0.147
Threshold Value	2.54	0.02	3.154	0.08
Threshold test *p*-value	0.065	0.00	0.165	0,00

^**^ = 5% significant level; and ^***^ = 10% significant level.

### Robustness analysis

To ensure the strength of the analysis of the suggested approach, the study formed a novel dataset characterized by [±10] and reproduced the findings. The findings within Table 9 depict a minute variance. Next, there was a different mediation impact on hotel corporate social responsibility and employee safety change. Specifically, safety compliance partly controlled the effect of corporate social responsibility on safety participation, and safety compliance and safety partaking mediated the impact on safety modification. The safety participation partially mediated the effect of safety compliance modification. The correlation among safety behavior dimensions was not evaluated in the past years.

**Table 9:** The green economic performance index.

**Country**	**2010**	**2011**	**2012**	**2012**	**2014**	**2015**	**2014**	**2015**
Brazil	0.54	0.57	0.51	0.58	0.64	0.59	0.58	0.64
China	0.94	0.98	0.94	1	1	1	0.98	0.97
India	0.41	0.59	0.49	0.38	0.62	0.47	0.57	0.58
Indonesia	0.58	0.57	0.39	0.58	0.57	0.58	0.41	0.46
Russia	0.44	0.44	0.38	0.69	0.65	0.39	0.48	0.49
Mexico	0.46	0.59	0.47	0.52	0.61	0.54	0.49	0.44
Turkey	0.58	0.45	0.48	0.54	0.57	0.69	0.47	0.48

This indicates the strength of the findings. The robustness of the results presents the obvious scientific analysis for media and public decision formulators (Table 10). This might cause an economic indication from the robustness analysis that the supply-aspect financing in a low carbon economy pathway is not anticipated to experience drastic expansion. Further, Mexico aims to aggressively reduce greenhouse gases pollution by 22% beneath the baseline scenarios in 2030, comprising a conditional cut of 26% beneath the baseline scenarios, whereas a reduced amount of 320 MtCO2eqy1 by 2050 is equally expected together with the extensive policy preparation, the above-granted aims warrant meaningful sectoral and social advancement (73).

**Table 10:** Threshold regression results.

**Variable**	**Model 1**	**Model 2**	**Model 3**
GF	0321^***^(0.0032)	0.0474^***^(0.0052)	0.0324^***^(0.0054)
REP	0.0189(0.0019)	0.0165(0.0065)	0.547^*^(0.0047)
HE	0.0625^*^(0.0458)	0.0624(0.0745)	0.0165(0.0411)
HCI	0.0003^**^(0.0001)	0.0025^**^(0.0005)	0.0001^***^(0.0005)
RandD	0.0248^**^(0.0032)	0.0428^***^(0.0074)	0.0165^***^(0.0065)
RE	0.0145^***^(0.0178)	0.0428^***^(0.0187)	0.0324^***^(0.0214)

* = 1% significant level;

** = 5% significant level; and

*** = 10% significant level.

Besides, the results for coal rent are said to be direct among all the witnessed quantiles, but solely mathematically meaningful within the three median quantiles. This shows that the introduction of mediated rent on coal spending in E-7 countries witnessed within the median quantiles grows ecological destruction between the E-7 countries. This finding contrasts sharply with the results from Nor and Masron (74), who discovered no causal association between conventional sources decreasing the level of CO2 pollution. Nevertheless, Peng et al. (75) and Zhang et al. (76) found different results with proof from Turkey.

Within the instance for the extra explanation, the analysis also found that certain regulatory measures on carbon pollution within the E-7 economies depicted a direct impact among the observed quantiles comprising coal, and the cost of damage depicted a direct impact among the witnessed quantile. This means that growing rent for coal spending tethers with the growing cost of carbon damage increases ecological destruction (77, 78).

## Conclusion and policy implication

This analysis applied the econometric approximation and entropy approach longitudinal data equation for the scenario of E-7 economies by utilizing panel data from 2010 to 2020. The findings depict that economic advancement can cause green economic destruction by influencing energy policy and industrial structure in varied ways. Moreover, green finance and public health expenditure are positively linked with green economic recovery, which confirms that higher public health expenditure increases countries' economic growth due to high efficiency and productivity of labor. Equally, the probability value of the one and dual-threshold equations passed the 1% analysis to summarize that there is a dual-threshold impact. The ordinary least square approximator findings from the regression approach directly impact the real gross domestic product on CO_2_ pollution. Hence, growing economic activity within the region due to manufacturing activities and implementing other complex generation processes have resulted in extra ecological destruction. Then, uncontrolled expansion is observed as a driver of ecological destruction in E-7 countries. In addition, RE has been discovered to attain an adverse effect on carbon dioxide pollution among the E-7 nations. This corroborates the switch of nations from conventional energy sources to renewable energy sources to fight increasing carbon dioxide pollution and satisfy the anticipation of increasing demand for energy resources.

This further means that ecological destruction can be minimal if extra energy can be supplied from nuclear energy sources among the E-7 nations. The findings for coal rent depict a direct and mathematical effect of coal rent on carbon pollution in E-7 economies. Nevertheless, the impact is common where coal rent spending is moderately charged. Equally, the cost of carbon damage depicts a directly meaningful impact on carbon pollution. This shows the growing carbon dioxide pollution within the E-7 countries due to the increasing economic activity worsening the pressure on the ecology. Thus, energy supply from crude, gas, and conventional sources is detected to gain the ecology, as the results depict an adverse effect on the environmental destruction among the E-7 countries. Ultimately, the analysis entailed some regular measures to gain insight into the impact of select energy programs on carbon dioxide pollution among the seven nations.

Hence, the approximated thresholds are 1.240 and 2.421, and green economic performance index score within the range of 0.54 to one is profound among the E-7 comparative analysis of the casualty and communication means in varied circumstances. Safety compliance partly mediated the effect of corporate social responsibility on safety participation, and safety compliance plus safety participation partly mediated the effect of corporate social responsibility on change. More so, safety participation partly mediated the effect of safety compliance on modification. The correlation among safety behavior dimensions had been past evaluated. The program allows businesses to generate RE to satisfy national goals set of 25% of RE supply by 2018, 30% by 2021, and 35% by 2024. This comprises a short-run goal of a clean energy supply of 5% in 2018 and 5.8% in 2019.

### Policy recommendation

Policies to motivate clean energy technology research and development are as follows:

1. To encourage green energy sources and green practices in operations—both of which have a significant positive impact on air quality due to their low carbon emission rates—regulatory bodies should provide tax exemptions and incentives for green technology.
2. Green practices should promote FDI inflows since they improve a country's image, attracting more FDI.
3. To maximize the deployment of quality improvements, economic policy should liberalize trade, increase FDI, and improve logistic services.
4. Heavy taxes and import charges with a financial penalty on non-green activities should be enforced by governmental authorities to discourage polluting automobiles and operations. On the other hand, the government should provide finance to the business sector to use renewable energy sources in its operations and purchase green vehicles, thereby decreasing healthcare costs and improving environmental sustainability and human health.
5. It is possible that “certification schemes,” a joint effort between the regulatory body and industry to improve green practices in operations, will aid in advancing a sustainable agenda.
6. To ensure efficient delivery of gains and subsidies, bureaucracy ought to be cut within the management of the structure of countries.
7. Energy management practices ought to be entailed to cut global dependence in a controlled and cost-effective way. It is worth stating that the success of these programs in solving the above parts of energy safeguards would determine their impacts on attaining energy security. Policy results must be detailed and implemented within this circumstance to satisfy these aspects' requirements.

## Data availability statement

The raw data supporting the conclusions of this article will be made available by the authors, without undue reservation.

## Author contributions

All authors listed have made a substantial, direct, and intellectual contribution to the work and approved it for publication.

## Conflict of interest

The authors declare that the research was conducted in the absence of any commercial or financial relationships that could be construed as a potential conflict of interest.

## Publisher's note

All claims expressed in this article are solely those of the authors and do not necessarily represent those of their affiliated organizations, or those of the publisher, the editors and the reviewers. Any product that may be evaluated in this article, or claim that may be made by its manufacturer, is not guaranteed or endorsed by the publisher.
